# 39.1 DNA METHYLATION OF IMMUNE CELLS IN PERSONS AT CLINICAL HIGH RISK FOR PSYCHOSIS

**DOI:** 10.1093/schbul/sby014.159

**Published:** 2018-04-01

**Authors:** Diana Perkins, Jeffries Clark, Jean Addington, Carrie Beardon, Kristin Cadenhead, Tyrone Cannon, Barbara Cornblatt, Daniel Mathalon, Thomas McGlashan, Larry Seidman, Ming Tsuang, Elaine Walker, Scott Woods

**Affiliations:** 1 University of North Carolina at Chapel Hill; 2 University of Calgary; 3 University of California, Los Angeles; 4 University of California, San Diego; 5 Yale University; 6 The Zucker Hillside Hospital; 7 University of California, San Francisco; 8 Beth Israel Deaconess, Harvard Medical Center; 9 Emory University

## Abstract

**Background:**

A dysregulated immune system is implicated in the development of psychotic disorders. Persons with schizophrenia have altered levels of circulating immune cell signaling molecules (cytokines), and elevation of specific cytokines predict conversion to psychosis in persons at clinical high risk. Whether these peripheral signals are a causal or a secondary phenomenon is unclear. But, subpopulations of circulating immune cells do regulate the brain from meningeal and perivascular locations influencing cognition, mood, and behavior, and thus may be relevant to schizophrenia vulnerability. Hematopoietic stem cells in the bone marrow differentiate into cascading subtypes depending on signals from other organs, especially the brain. For example, a monocyte subpopulation emerges with repeated social defeat that establish the persistence of anxiety-like behaviors; blocking their release or inhibiting their attachment to brain vascular endothelium prevents the emergence of anxiety-like behaviors. In humans, a similar monocyte subpopulation is associated with social isolation and other adversities including low SES, chronic stress, and bereavement.

**Methods:**

The North American Prodrome Longitudinal Study (NAPLS2) is an eight-site observational study of predictors and mechanisms of conversion to psychosis The full cohort includes 763 at clinical high risk (CHR) based on the Criteria of Prodromal State (COPS) and 279 demographically similar unaffected comparison (UC) subjects. Methylation of whole blood DNA collected in PAXgene tubes at baseline was analyzed with the Illumina 450k array in a subgroup of 59 subjects who converted to psychosis (CHR-C), 84 CHR subjects followed for 2 years who did not develop psychosis (CHR-NC) and 67 unaffected subjects (UC). Our analyses focused on methylation of promoter regions of genes, associated with gene expression. Classifier construction used Coarse Approximation Linear Function (CALF) with bootstrapping of 1000 random 80% subsets with replacement to determine statistical likelihood.

**Results:**

We found highly overlapping sets of differentially methylated promoter regions in CHR-C subjects compared to CHR-NC and to UC subjects. A set of 10 markers correctly classified CHR-C and CHR-NC subjects with high accuracy (AUC=0.94, 95% CI 0.89–0.98). Included was SIRT1, a gene that is upregulated with HSV reactivation.

**Discussion:**

Circulating immune cells excerpt powerful influences on mood, cognition and behavior. An obvious example is the experience of most human with “sickness syndrome”, characterized by apathy, avolition, and withdrawal, and triggered by immune-cell-released cytokines producing an adaptive, resource conserving, behavioral response. While at an early stage, our findings further implicate immune system dysregulation as a mechanism in the development of psychosis.

